# Internal crack characteristics in very-high-cycle fatigue of a gradient structured titanium alloy

**DOI:** 10.1038/s41598-020-61484-3

**Published:** 2020-03-16

**Authors:** Xiangnan Pan, Guian Qian, Shengchuan Wu, Yanan Fu, Youshi Hong

**Affiliations:** 1 State Key Laboratory of Nonlinear Mechanics, Institute of Mechanics, Chinese Academy of Sciences, Beijing, 100190 China; 2 School of Engineering Science, University of Chinese Academy of Sciences, Beijing, 100049 China; 3 State Key Laboratory of Traction Power, Southwest Jiaotong University, Chengdu, 610031 China; 4 Shanghai Synchrotron Radiation Facility (SSRF), Shanghai Advanced Research Institute, Chinese Academy of Sciences, Shanghai, 201204 China

**Keywords:** Engineering, Materials science

## Abstract

Gradient structure (GS) is commonly designed and processed in engineering materials to improve mechanical properties especially fatigue performance by taking advantage of the strengthened surface. However, whether the very-high-cycle fatigue (VHCF) property can be improved by GS is questioning due to the different crack initiation mechanisms between low-, high-cycle and VHCF. In this paper, GS of a Ti-6Al-4V alloy is generated by pre-torsion and characterized by electron backscatter diffraction. Then the VHCF behavior of the GS specimen is studied. The fractography and synchrotron radiation X-ray microtomography presented detailed characteristics of the internal crack initiation region in VHCF of the titanium alloy with GS. The results indicated that, in contrast to the low- and high-cycle regimes, the VHCF strength is reduced for the specimens with GS. Thus, the GS induced by pre-torsion cannot enhance the VHCF strength of the titanium alloy. This implies that VHCF test (property) is an important consideration for the microstructural designed materials. The graphical abstract is available in Supplementary information.

## Introduction

Fatigue damage causes the failure of more than 90% engineering materials and structures. According to the traditional knowledge, fatigue crack initiates from the surface of material^1^. Therefore, many surface strengthening methods, such as surface mechanical attrition or grinding treatment, surface quenching, shot peening, etc., have been widely used as counter strategies to improve the fatigue resistance of the components by introducing the refined microstructure with gradient distribution of grain size and residual compressive stress^2,3^. It is known that crack is hard to initiate from small grains, and the residual compressive stress may enhance the fatigue strength^1,2^. In recent years, pre-torsion method was used to produce gradient structure (GS) in metallic materials owing to its low cost and easy processing^4–7^. During the pre-torsion process, grain size can be substantially refined due to severe plastic deformation (SPD)^8^, and the GS is generated by the gradient strain. A number of experimental results^9,10^ confirmed that the layer of GS improves fatigue property in low-cycle-fatigue (LCF) and high-cycle-fatigue (HCF) regimes, i.e. for the cases that the number of fatigue failure cycles is lower than 10^7^. However, with the development of modern civilization, there is practical demand of engineering structures for more than 10^7^ loading cycles of safe performance. Thus, very-high-cycle fatigue (VHCF) has become an important topic, and has received increasing attention^11–13^. However, VHCF test is very time consuming, e.g. it takes 4 days to reach 10^7^ cycles and more than 1 year for 10^9^ cycles with conventional frequency of 30 Hz via a servohydraulic system. This limits the VHCF research and hence the VHCF behavior is still rarely studied for metallic materials with a designed microstructure, such as GS, heterogeneous, hierarchical, etc. Thus, whether the VHCF fatigue property can be improved by the layer of GS is still questioning as the crack initiation mechanism in VHCF differs from that in LCF and HCF regimes^11,13–15^. Hence, this paper aims to investigate the VHCF behavior of a Ti-6Al-4V alloy with a GS layer. As an accelerated testing method, ultrasonic cycling with resonant frequency 20 kHz is employed for the VHCF test, which takes about 8 minutes to reach 10^7^ cycles and 14 hours to 10^9^ cycles. The use of ultrasonic frequency may cause loading frequency effect resulted from the increased strain rate and the induced temperature rise of the tested specimen. Many experimental results^14–17^ have shown that this effect may cause the change of fatigue strength, and it has little effect on crack origin mode (e.g. surface or interior) of fatigue failure. In fact, for titanium alloys^14,15^, there is no evident difference between ultrasonic (20 kHz) fatigue data and conventional frequency (30 Hz) tests by servo-hydraulic machines in HCF and VHCF regimes.

## Material and Methods

**Figure 1:**
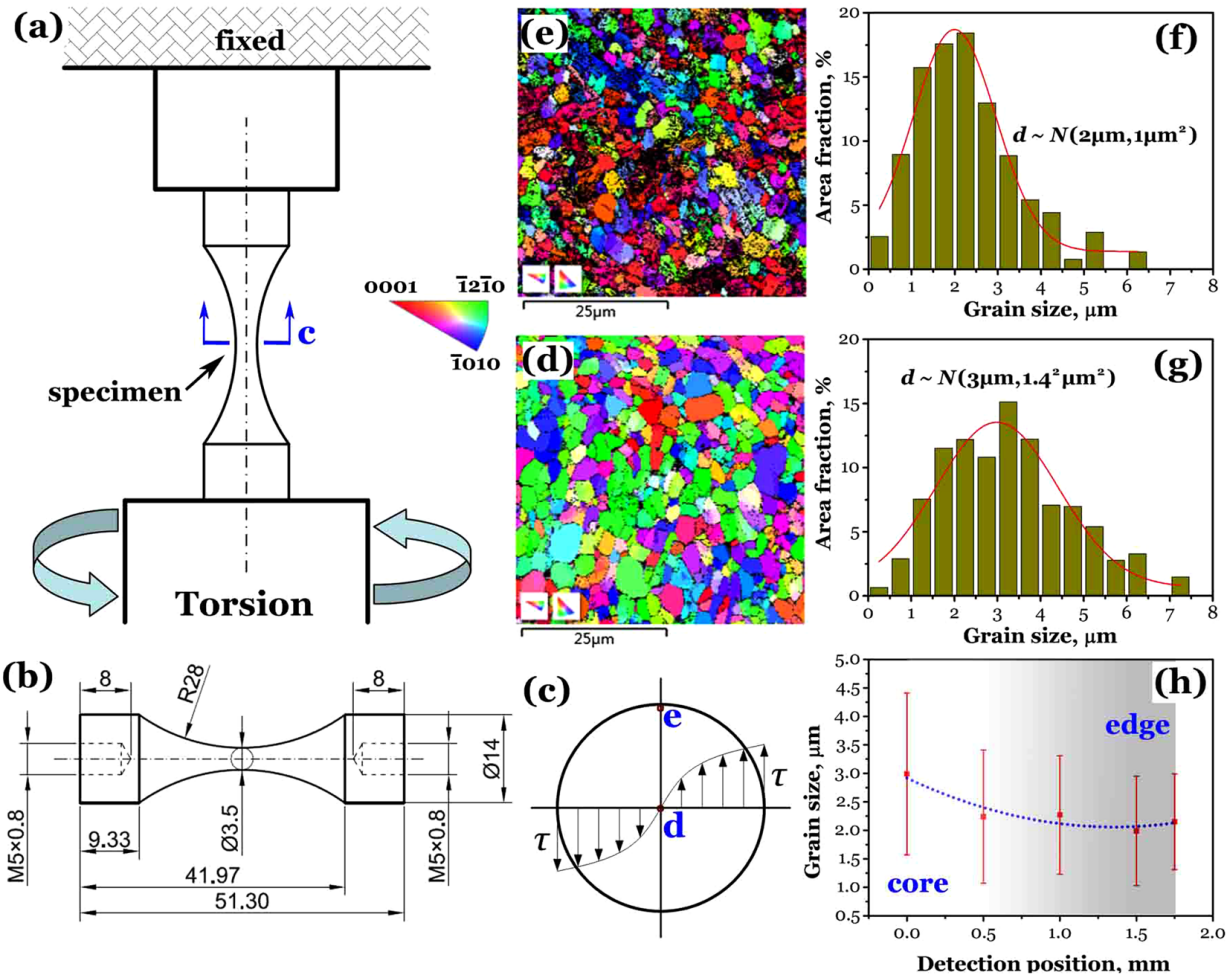
Gradient structured specimen produced by torsion: (**a**) pre-torsion setup, (**b**) specimen shape and dimensions (in mm), (**c**) shear stress distribution in the cross section c of specimen, (**d**,**e**) EBSD images of IPF at d and e in (**c)**, (**f**,**g**) Gauss distribution of grain size at point e and d, and (**h**) variation of grain size along specimen radius from d to e in cross section c.

A titanium alloy Ti-6Al-4V was used in this study with the chemical composition (wt.%) of 6.37 Al, 4.24 V, 0.09 Fe, 0.017 C, 0.015 N, 0.0015 H, 0.11 O and balance Ti. The as-received (as-rec) material with equiaxed microstructure (EM) was processed to let GS be generated in the specimens by pre-torsion (pre-tor). Figure 1a,b schematically present the method and the specimen dimensions. Figure 1c illustrates the shear stress distributed in the minimum cross section of the specimen. The applied torque caused great gradient strain that plastically deformed the edge region and elastically deformed the core region in the cross section. In the pre-tor process, an as-rec specimen was twisted at a rate of 5 degree/minute until the torque reached 8 kN·m, hold for 5 minutes, and then the specimen was unloaded.

Microstructure characterizations for the core region and the edge region are displayed in Fig. 1d,e, which show the grain orientation of IPF (inverse pole figure) examined by electron backscatter diffraction (EBSD) mapping via a field emission scanning electron microscope (FE-SEM) equipped with a NordlysNano detector. Based on the EBSD results, normal distributions for grain size *d* as *N* (mean value: 2 μm, variance: 1 μm^2^) at the edge region and *N* (3 μm, 1.4^2^ μm^2^) the core region are fitted from the statistical column bars of area fraction in Fig. 1f,g, respectively. Further, three additional positions along the radius of the EBSD sample from core d to edge e (Fig. 1c) were examined, and the Gauss parameters of grain size distribution were readily obtained as shown in Fig. 1h, which presents the distribution tendency of grain size in the cross region of the specimen with GS. It is experimentally confirmed that the GS with the distribution of grain size and residual strain is generated in the pre-tor process. For comparison, the EBSD characterization for the as-rec specimens exhibited the same normal distribution of grain size *d* as *N* (3 μm, 1.4^2^ μm^2^) and the similar orientation map of IPF as shown in Fig. 1d,g. This indicates that the core region of the minimum cross section of either the pre-tor or as-rec specimen is of the similar EM with the mean grain size of 3 μm and its variance of 1.4^2^ μm^2^.

The ultrasonic fatigue test was conducted for both as-rec and pre-tor specimens with fully reversed tension-compression cycling (stress ratio *R* = −1) via a Lasur GF20-TC at room temperature and in air. The cyclic loading direction is parallel to the longitudinal direction of the as-rec bar of the material. During the test, every specimen was cooled by compressed cold air to avoid temperature rise of the specimen thus to reduce the frequency induced thermal effect.

Although the fractograph records important information about fatigue failure, fractography is a post-testing observation method, which is impossible to depict the ongoing damage process of VHCF. Thanks to the superior penetrating ability, the *in-situ* test method of high-energy X rays has been used to image the defects and cracks inside the metallic materials^18^. The internal morphology can be reconstructed by the computer tomography (CT) technique. In this paper, several pre-tor specimens experienced VHCF were selected for the purpose of capturing internal crack behavior, which was performed at the BL13W1 of Shanghai Synchrotron Radiation Facility (SSRF). A tensile force of 0.5 kN was applied to keep the cracks still open during the CT scanning by the *in-situ* rig developed by Wu *et al*.^19–21^.

## Results and Discussion

**Figure 2:**
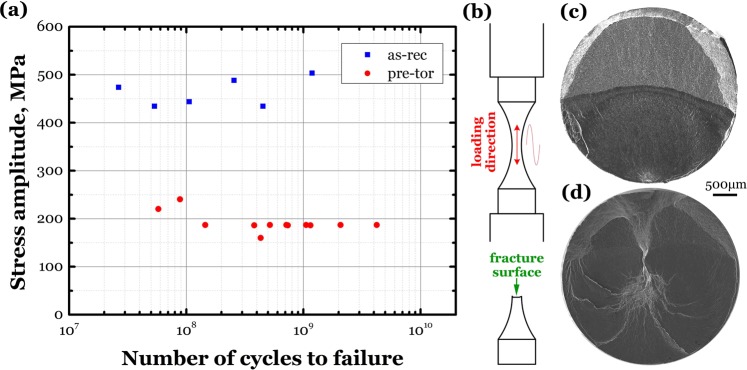
(**a**) S-N data at stress ratio *R* = −1 for two types of specimens, (**b**) schematic illustration of loading direction and fracture surface, (**c**) surface crack initiation inducing VHCF of as-rec Ti-6Al-4V alloy with EM, *σ*_a_ = 474 MPa, *N*_f_ = 2.64 × 10^7^, and (**d**) internal crack initiation inducing VHCF of gradient structured titanium alloy, *σ*_a_ = 187 MPa, *N*_f_ = 2.07 × 10^9^.

Figure 2a shows the obtained S-N data of the as-rec and pre-tor specimens, and Fig. 2b schematically shows the cyclic loading direction with the fracture surface to be examined. The present fatigue testing results of Fig. 2a indicates that the as-rec specimens failed in the range between 2.64 × 10^7^ and 1.19 × 10^9^ cycles under stress amplitude *σ*_a_ between 434 and 503 MPa, and the pre-tor specimens failed in the range between 5.78 × 10^7^ and 4.23 × 10^9^ cycles with the fatigue strength *σ*_a_ = 187 MPa when the failure cycles *N*_f_ > 5 × 10^8^. It is obvious that the VHCF property is markedly degraded for the tested material with GS, meaning that the existence of GS is not an efficient way to improve the VHCF property. In the literature, it was reported that pre-tor treatment is of benefit to monotonic and cyclic performance in LCF and HCF regime. For instance, refs. ^4–7^ showed that pre-torsion enhances the tensile property and fatigue strength in LCF and HCF regime of steels, ref. ^10^ showed that pre-torsion increases the tensile strength of pure titanium, and ref. ^9^ showed that the GS improves the LCF and HCF resistances of a Ti-6Al-4V alloy. The present result contradicts to the previous understanding that fatigue property can be improved by the introduction of GS into the material.

Figure 2c,d show the typical VHCF fractographs for as-rec and pre-tor specimens, respectively, by using scanning electron microscopy (SEM) with a JEOL JSM-IT300. Two failure modes dominating the VHCF behavior are clarified: surface crack initiation for as-rec and internal crack initiation for pre-tor specimens, respectively.

**Figure 3:**
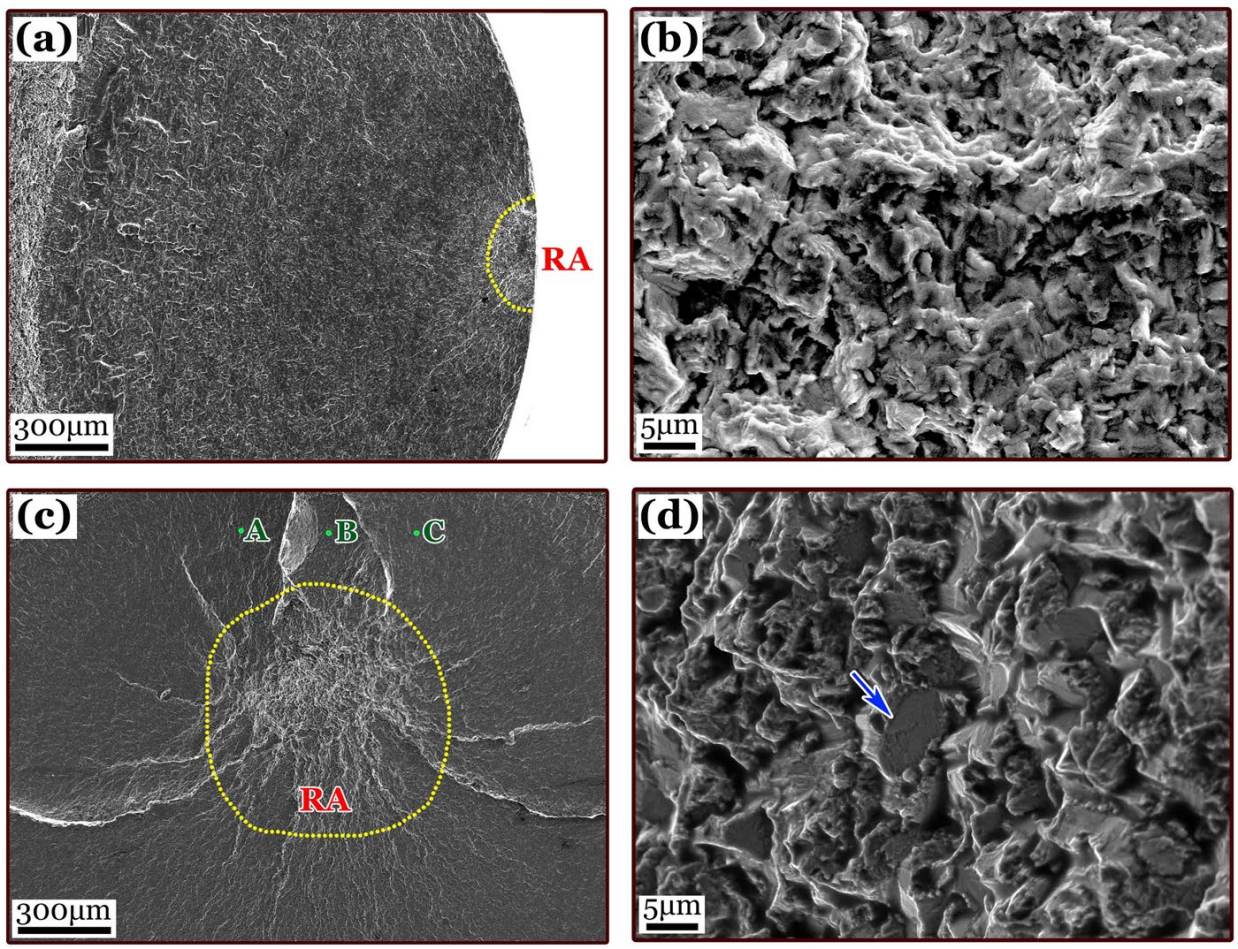
SEM images showing RA morphology of VHCF crack initiation region on fracture surface: (**a**) low and (**b**) high magnification for a surface case of as-received specimen, *R* = −1, *σ*_a_ = 444 MPa, *N*_f_ = 1.06 × 10^8^; and (**c**) low and (**d**) high magnification for an interior case of pre-torsion specimen, *R* = −1, *σ*_a_ = 187 MPa, *N*_f_ = 4.23 × 10^9^; the arrow in (**d**) indicating a large cleavage facet.

Figure 2d shows a big rough area (RA) of crack initiation region with the projected area size of about 1 mm in diameter, located near the center of the fracture surface with a “spider” morphology. It is known that the RA region is the characteristic zone of crack initiation for titanium alloys with EM failed in HCF and VHCF regimes^22–24^. The RA morphology also existed in the surface of as-rec material subjected to VHCF. Figure 3a illustrates one case of surface RA morphology and Fig. 3b presents its high-magnified detail using FE-SEM via an FEI Quanta 200 FEG, whereas Fig. 3c shows a case of the “spider” shaped internal crack initiation with RA region in the pre-tor specimen failed at 4.23 × 10^9^ cycles and *σ*_a_ = 187 MPa. By comparing Fig. 3c with 3a, the RA region in the pre-tor specimen has a larger size than that in the as-rec one. The former size is about 700 μm and the latter is about 300 μm in the projected plane of fracture surface. In contrast to the semi-circular of “fish-eye” morphology induced by one surface crack in as-rec specimen with relative flat fracture surface, the internal cracked fracture of pre-tor specimen consists of multiple crack growth planes. Specifically, in Fig. 3c, the bright ridges of the “spider legs” are the intervened marks met by the multiple internal grown cracks, with points A, B and C indicating different crack planes. In addition, Fig. 3d gives a detailed morphology of the RA surface showing a big cleavage facet about 9 μm in equivalent diameter, which is 3 times of the average grain size of 3 μm in the original pre-tor specimen.

It is obvious that the transition of crack initiation site from specimen surface to interior in VHCF regime will induce the change of fatigue strength for the as-rec and pre-tor specimens. The EBSD result of Fig. 1e and 1f shows that the grain size refinement occurs in the edge region of the pre-tor specimen and the declining EBSD resolution (dark areas in Fig. 1e) indicates the presence of residual strain. The refined grains and residual compressive stress inhibit the VHCF crack initiation, and thereby no crack initiates from the surface or within the edge region of the pre-tor specimen. The EBSD result of Fig. 1d and 1g shows that the grain size distributed in the core region of the pre-tor specimen is basically the same as that of as-rec specimen, so the cleavage strength of a single α grain should be equivalent to that of the as-rec one. Nevertheless, the residual deformation caused by pre-tor makes the core region of the specimen to be slightly twisted by a residual torque, and to contain locally residual tensile stress, thereby reducing the threshold value of cleavage formation. As shown in Fig. 3c,d, the large number of cleavage facets are also the evidence to support this, which is similar to the RA morphology of the VHCF crack initiation region in titanium alloys under the cases of stress ratio *R* > 0^23,24^.

**Figure 4:**
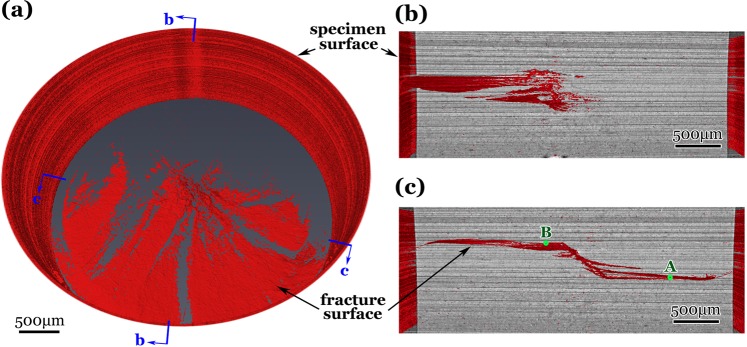
Synchrotron radiation X-ray microtomography (SR-μCT) for internal crack in an unbroken pre-torsion specimen experienced VHCF, *R* = −1, *σ*_a_ = 187 MPa, *N*_f_ = 1.05 × 10^9^, (**a**) projection image of fracture surface before broken, (**b**) tilted view of cross section b in (**a**), and (**c**) tilted view of cross section c in (**a**); note that (**b**,**c**) being the projection images containing all crack information beyond the mentioned cross section.

A typical micrograph of synchrotron radiation X-ray microtomography (SR-μCT) is shown in Fig. 4 for a pre-tor specimen experienced 1.05 × 10^9^ cycles under *σ*_a_ = 187 MPa with *R* = −1. The morphology of internal crack demonstrates that the RA region has a spread size of about 500 μm in the loading direction (Fig. 4b,c). The RA region was formed by many isolated and converged cracks that originated from the specimen interior and grew outwardly. The “spider legs” are the connections of associated crack planes, which are the cracks originated from different heights of the specimen as points A and B indicated in Fig. 4c.

In summary, the detail characteristics of the internal crack initiation region in VHCF of a titanium alloy with GS were first reported via SR-μCT at SSRF and SEM. The internal crack initiation with big RA region that has a size of more than 700 μm on the projected fractograph and span about 500 μm in the loading direction causes the decrease of VHCF resistance in the pre-tor specimen of Ti-6Al-4V alloy with gradient structured EM. The GS as well as residual stress induced by pre-tor cannot enhance the VHCF strength of the titanium alloy. It is suggested that the metallic materials with GS may not be able to improve the VHCF performance due to the transition of crack initiation mode from specimen surface to interior, even if they have superior mechanical properties in monotonic tension, LCF and HCF. Therefore, the VHCF evaluation should be taken into account in the design of structural materials that are expected for a long service life.

## Supplementary information

Supplementary information.

## Data Availability

The data that support the findings of this investigation are available from the corresponding author upon reasonable request.
