# Supplementary material for: Internal crack characteristics in very-high-cycle fatigue of a gradient structured titanium alloy
Source: Sci Rep. 2020 Mar 16;10:4742. doi: 10.1038/s41598-020-61484-3 (PMC7075889; doi:10.1038/s41598-020-61484-3)

## Internal crack characteristics in very-high-cycle fatigue of a gradient structured titanium alloy

Xiangnan Pan^a,b^, Guian Qian^a,b^, Shengchuan Wu^c^, Yanan Fu^d^, Youshi Hong^a,b^*

^a^ State Key Laboratory of Nonlinear Mechanics, Institute of Mechanics, Chinese Academy of Sciences, Beijing 100190, China

^b^ School of Engineering Science, University of Chinese Academy of Sciences, Beijing 100049, China

^c^ State Key Laboratory of Traction Power, Southwest Jiaotong University, Chengdu 610031, China

^d^ Shanghai Synchrotron Radiation Facility (SSRF), Shanghai Advanced Research Institute, Chinese Academy of Sciences, Shanghai 201204, China

*Corresponding author, Email: [hongys@imech.ac.cn](mailto:hongys@imech.ac.cn)

## Graphical Abstract

Internal crack initiation induced very-high-cycle fatigue for a gradient microstructure


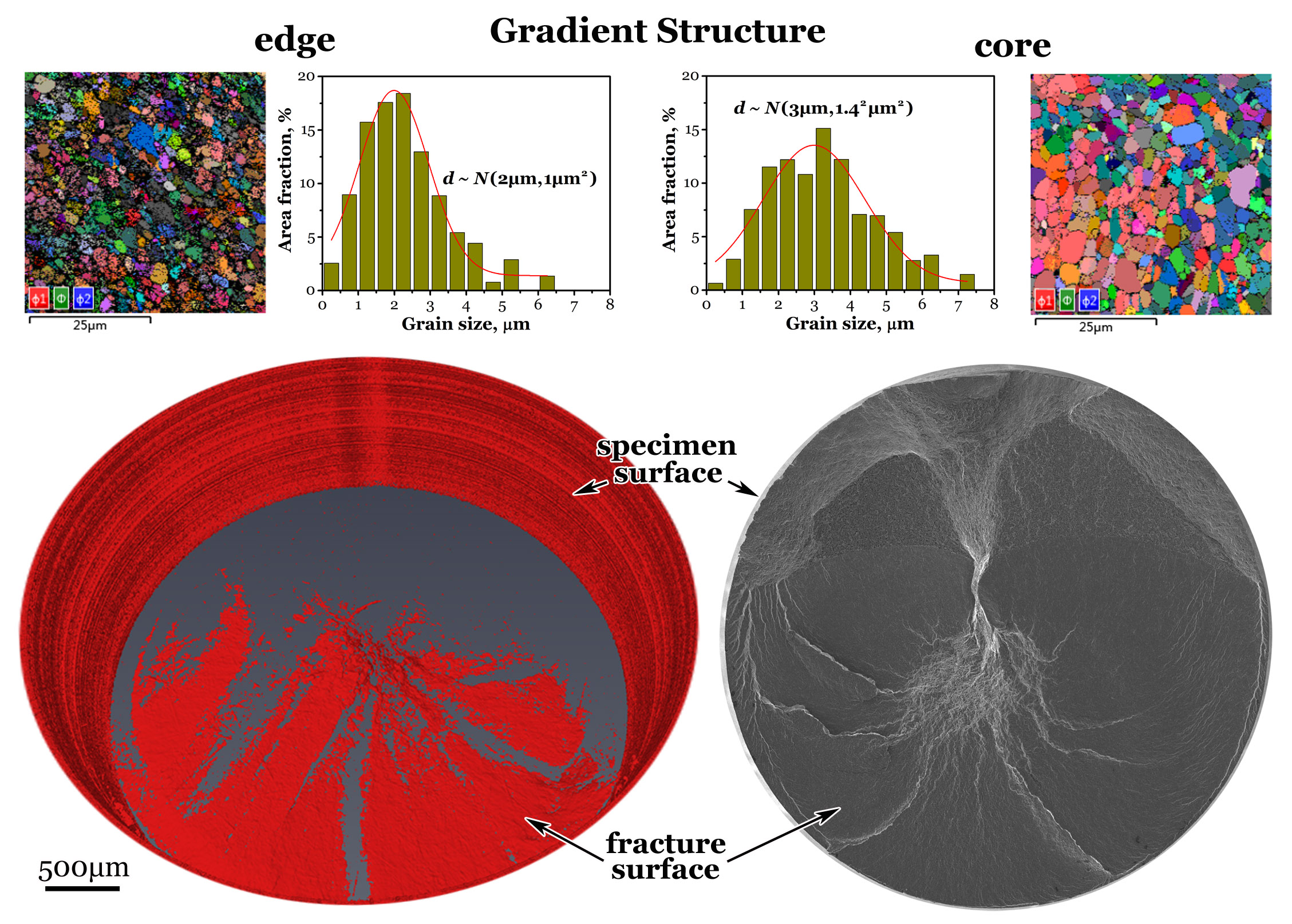

Supplement: Supplementary file 1 — Supplementary information. [file 41598_2020_61484_MOESM1_ESM.doc]
